# Characteristics of the Optimal Cognitive Behavioral Analysis System of Psychotherapy (CBASP) Therapist Role

**DOI:** 10.3389/fpsyt.2020.609954

**Published:** 2021-01-18

**Authors:** James P. McCullough

**Affiliations:** Emeritus Professor of Psychology, Virginia Commonwealth University, Richmond, VA, United States

**Keywords:** interpersonal psychotherapy, persistent depressive disorder, therapist role, disciplined personal involvement, cognitive behavioral analysis system of psychotherapy (CBASP)

## Abstract

The characteristics of the optimal CBASP therapist role for the treatment of the Persistent Depressive Disorder patient (chronic depression) is delineated in this paper. This paper contains the opinions and experiences of the creator of CBASP who has developed and revised the model over more than 4 decades. The paper is not a rigorous study nor a review of rigorous studies. The difficulties of the patient are briefly discussed and then the characteristics of the optimal clinical role are presented. The clinical role of CBASP, the only model to have been developed specifically to treat the chronically depressive patient, is unique in the field of psychotherapy. Four role categories describing the behavior of the best therapists are presented and discussed: (1) the therapist is able to enact a Disciplined Personal Involvement clinical role with the patient; (2) the therapist is able to implement an acquisition-learning approach to therapeutic administration; (3) the practitioner is able to adhere to the standards of CBASP technique administration; and finally, (4) the clinician is able to implement several facilitative interpersonal skills.

## Introduction

Becoming a successful CBASP psychotherapist is not a simple undertaking. The reasons are two-fold: first, the early-onset Persistent Depressive Disorder patient (PDD) (1) presents unique and difficult challenges to practitioners, and secondly the CBASP clinical role is qualitatively unique given its combination of techniques and therapist role requirements. Chronic patients are difficult because of the entrenched cognitive-emotional-behavioral patterns many patients bring to treatment. They orbit in a trajectory of overlearned interpersonal-avoidance due to toxic developmental histories. Secondly, the patient's unique pathological and long-standing disorder requires practitioners to actualize a personal relationship that seeks to modify a primitive lifestyle. CBASP practitioners are trained to enact a clinical role which adds a “humanizing experience” to the patient. They become personal comrades to individuals who, more than likely, never had one—a friend-relationship where trust, support, and caring characterize the encounter. This clinical role, labeled *Disciplined Personal Involvement*, sets the CBASP therapist role apart from many other therapist role models in the field today. Other therapeutic models traditionally require clinical role behavior that precludes personal involvement. Personal attachments with patients have been labeled taboo or verboten (2, 3).

For almost half-a-century, I have studied the taxonomy of chronic depression and treated the chronically depressed patient—even before we had a diagnostic category to describe this disorder (4). As a university faculty member, I studied and focused on the treatment of chronic depression for almost fifty years. Participating in four national clinical trials conducted at 12 university sites, I served as Principal Investigator for my site as we randomized 2,200 chronically depressed outpatients in medication and psychotherapy investigations. In one trial, we reported the highest response rates ever recorded for the PDD-D patient (77%) in the combination CBASP and medication cell (5). In addition, I've treated ~450 PDD outpatients in my career (6), and I was secondarily involved in the mood disorder revisions in *DSM-5* (1) where the first chronic depression category appeared as an independent taxonomy. PDD was no longer classified in *DSM-5* as a “specifier” for major depression. Lastly, I created the only psychotherapy model, Cognitive Behavioral Analysis System of Psychotherapy (CBASP) (6–10), that was developed specifically to treat the PDD disorder. Over the years, I have trained hundreds of professionals to administer CBASP. Retiring in 2017 from Virginia Commonwealth University, I feel qualified to comment below on the optimal characteristics of successful CBASP psychotherapists. I turn now to the early-onset PDD patient who, as stated above, is one of the most difficult outpatients we see in clinical practice.

## The Persistent Depressive Disorder-Dysthymia Patient

### Social Dysfunction

During the training workshops I have conducted over the years, I asked the participants to list the characteristics that describe the early-onset PDD-D patients they have treated. The list frequently includes most of the following features (6):

- Traumatic developmental history involving either sexual, physical, and/or emotional abuse, or physical or emotional deprivation
- Little to no motivation to change one's behavior
- Pervasive social avoidance
- Generalized interpersonal withdrawal and detachment
- Thinks and talks in a primitive-illogical manner about interpersonal relationships
- Unable to generate interpersonal empathy
- Anger toward one or more significant others
- Overwhelming feelings of helplessness and hopelessness
- Generalized pessimistic view that nothing can ever be different
- Pervasive feelings of inadequacy
- Feeling guilt about the state of one's life
- Behavioral passivity
- Pervasive negativity
- Feeling unlovable and that no one could ever care for one
- Generalized feelings of being a failure
- Strong expectancy of interpersonal rejection
- Suicidal ideation that may also include actual suicide attempts

Sitting with a patient who embodies these characteristics pulls predictable interpersonal and counter-transference reactions from many psychotherapists (6). Workshop participants who have treated these patients have no difficulty listing the effects patients have on them:

- A noticeable feeling of interpersonal loneliness
- Feelings of incompetence and hopelessness when patients continue to complain that nothing they do matters
- Feelings of being “put in a rejection box” and interpersonally pushed away by the detached style of the patient
- Feelings of being frustrated and angry by the person's apparent lack of any motivation and by their pervasive interpersonal avoidance patterns
- Becoming tired, drained, and worn out—feeling that I am trying to pull a “dead weight” during the session
- “I want to quit seeing this individual and must force myself to continue treatment”
- “When patients tell me that no one likes them, I think they are correctly reading others—no one could like them!”

### Etiology of Early-onset PDD

Etiological events in the histories of PDD patients derail normal social-emotional maturational development and entrap the child and adolescent in a preoperational state of development (11). All of the following clinical researchers [e.g., Spitz (12), “failure to thrive” researchers (e.g., (11, 13–17)), “paroxysms” which disrupt normal cognitive development] suggest that excessive emotionality, adverse familial circumstances of long duration, and severe neglect or trauma may interfere with normal cognitive-emotional maturation and physical development. Such events may also derail or retard normal developmental processes. A child's living environment, when it becomes an obstacle course with no resolution, inhibits normal growth and maturation. One characteristic of maturational derailment is suggested when adult PDD patients report an early-onset Dysthymia condition co-morbid with the chronic depression diagnosis (6, 10, 18). Under such circumstances, surviving the “hell of the family,” not normal growth-directed behavior, becomes the child's only developmental goal (19). The hallmark emotions of chronic depression—helplessness and hopelessness—are appropriate and valid symptoms associated with a familial world that offers “no exit” (20). The categories of maltreatment often reported are *emotional mistreatment, parental loss, physical abuse, sexual abuse*, and *physical neglect* (21, 22). Frequently, early-onset patients bring the “results” of a catastrophic developmental history into treatment and present a difficult challenge to psychotherapists (e.g., extreme interpersonal detachment and withdrawal; pervasive withdrawal in interpersonal challenges, etc.).

### Preoperational Functioning Among Early-onset PDD Adults

A unique picture of psychopathology unfolds as one listens carefully and observes the way chronic patients talk and behave. The individual is isolated interpersonally, talks in a monologue manner using a well-rehearsed script of rejection, and lives in quiet despair within a self-contained world that is not informed by external influences. Nothing new enters and nothing leaves this phenomenological orbit. The patient presents with a terrible sense of “sameness.” Existentially, the patient describes a lifestyle where time appears to have stopped—*the present reflects the past and the future bodes only more of the same*. (9) labeled this temporal outlook as a “snapshot view of reality.” These internal snapshots of rejection and hurt are *freeze-framed* in the patient's brain as evidenced by the chronicity of the PDD disorder (6, 19).

Piaget's (11, 23, 24) second structural stage of maturational development, *preoperational functioning*, appropriately describes the cognitive-emotional functioning level of many early-onset PDD patients. The patient is dominated by the immediacy of experience. (a) Patients think in a *precausal* and *prelogical* manner, drawing conclusions about the external world, jumping from a premise to a conclusion without any hypothesis testing—the external world of others is the way it is simply because patients believe it to be so. Reasoned viewpoints of others have no informing effect on this entrenched perceptual outlook. The logical and causal strivings of therapists, at least at the outset of treatment, usually fail to modify this primitive cognitive outlook. An example of such illogic is illustrated in one adult patient's report concerning her experience at a company picnic:

*Patient: Company photographer didn't take my picture at the company picnic. He took Susan, Jane, and Phyllis' pictures but not mine. He didn't take my picture because he doesn't like me*.

Therapist: Did you ask him to take your picture?

*Patient: It wouldn't have mattered. He would not have done it because he doesn't like me*.

Therapist: What evidence do you have for this assumption? How do you know he doesn't like you?

*Patient: I've never asked him. I don't have to. I just know he doesn't like me*.

(b) A pervasive *egocentric lifestyle* also characterizes the patient. All roads lead to the self. When listening to new patients' verbalizations, one rarely hears comments that shift the attentional focus away from *I, me*, and *my*. (c) Another preoperational characteristic is the inability *to generate interpersonal empathy*. Emotional sensitivity to interpersonal rejection must not be confused with empathy. Empathy generation requires abstractive ability and the beginning patient does not possess this in the interpersonal-social realm. Abstractive thought or the ability to disengage from the present situation and take a step back to consider alternatives is not an option.^1^ Adept CBASP therapists will produce an observable maturational shift in cognitive-emotive functioning over the course of treatment that will enable the person to gain control of the PDD condition and move toward remission and maturity.

(d) The ability *to regulate one's emotional life* is non-existent at therapy outset. Emotional regulation requires the presence of an abstractive capability which the preoperational patient does not possess. As suggested above, to overthrow the “snapshot view of reality” requires the individual to be able to perceptually disengage from the immediacy of the moment and consider alternative strategies in a planful, problem-focused manner (25).

CBASP therapists move their patients from preoperational levels of functioning to formal operational (abstractive) levels by systematically exposing them to in-session behavioral consequences. Maturational shifts in treatment are well-documented in the Piagetian therapeutic literature [e.g., (26, 27)]. The CBASP construct of *perceived functionality* denotes this acquired maturational shift when the patient can identify the environmental consequences of their behavior—the attainment of *perceived functionality* suggests that the individual has reached a formal operations level of thinking.

Summarily, the etiology and preoperational levels of functioning make the PDD patient a significant and unique challenge. The refractory nature of the disorder, the etiology of a prolonged and toxic developmental upbringing which has produced a maturational derailment, the interpersonal fear-avoidance of the patient due to a history of maltreatment, the perceptual disconnection from one's social-interpersonal environment which inhibits the possibility of behavioral change, taken together, require a *clinical role* qualitatively different from the traditional roles. To say that the PDD patient is different vis-a-vis other patient types is an understatement! The patient *IS* different and, put in more frank terms, the early-onset, adult PDD patient enters psychotherapy functioning at the cognitive-emotional maturity level of a 4–6-year-old child. The most outstanding CBASP therapists appreciate the immaturity of this patient and do not overestimate the learning potential of the patient; instead, they adjust their teaching behavior accordingly.

## Optimal Therapist Role Characteristics

The best and most effective CBASP psychotherapists I've worked with over the years evince characteristics that fall into four general categories: (1) Able to enact a Disciplined Personal Involvement clinical role with the patient; (2) Able to implement an acquisition-learning approach to therapeutic administration; (3) Able to adhere to the standards of CBASP technique administration; (4) Able to implement several facilitative interpersonal skills.

### Able to Enact a Disciplined Personal Involvement Clinical Role

Disciplined Personal Involvement [DPI: (10)] is based upon the Kieslerian concept of interpersonal interaction (28–30). Therapists who master DPI create salubrious *person* [therapist] x *person* [patient] interactions with patients in all that they do. Perceiving relationships through an interpersonal lens requires these practitioners to implement an extreme *empathetic perspective*. From the clinician's perspective, there is always a reciprocal relationship between speaker and hearer from the first moment a patient steps into the office. But, at the outset of treatment with the chronically depressed preoperational patient, it is the therapist who works from this empathic perspective—not the patient. However, by the end of treatment, the generation of empathy will become a reciprocal activity for both, because the patient will have learned from the therapist how to function empathically.

DPI also requires that one becomes adept at using the Impact Message Inventory (IMI) (28–30), an empirical instrument that measures the intensity of the impact messages of the patient. The best CBASP therapists are able to interpret the interpersonal “impacts” patients have on them, they are able to diagnose the interpersonal functioning of the patient from these impact messages and ultimately, they use this impact message information to teach patients to behave with them and others in more adaptive ways. Information describing how CBASP therapists utilize the IMI during therapy may be found in McCullough [(10), pp. 23–30]. These practitioners always directionally begin their in-session work *from themselves outward to the patient*—meaning they introspectively know the patient's impacts on them before they take the next step in the session.

The DPI role in CBASP is also the most misunderstood component of the model and the best CBASP therapists avoid misunderstanding DPI. What is meant here is that many CBASP-trained clinicians continue to speak of DPI as a “technique” to be administered. For example, some will say, “I am now doing DPI.” DPI is *NOT* a technique! Rather, it describes *the way* CBASP therapists always relate interpersonally to patients. *DPI is a clinical style to be lived out in the session with the chronic patient*. Relating to the individual in a DPI style is directly related to achieving the first goal of CBASP—that is, the creation of *felt dyadic safety* within the relationship. Patients entering treatment and who report an abusive history as children are fearful and avoidant of interpersonal engagement, opting to remain inhibited and withdrawn. The DPI style denotes one's willingness to be a comrade with a person who, more than likely, never had one. It does not mean that therapists and patients become drinking buddies, business partners, date, sleep together, share gossip, meet for coffee after work hours, or become chat room pals. Rather, personal involvement describes the optimal practitioner's style that is grounded upon the well-established learning principles of Skinner (31, 32). This style is used to choreograph personal reaction contingencies (personal responsivity) in the session so patients learn new associations. In choreographing in-session learning contingencies (of which more will be said in a moment), the personal involvement style also utilizes Albert Bandura's concepts of imitation learning and modeling (33). Bandura notes that in many languages, the word for “teach” is the same as the word for “show,” and the synonymity is literal in DPI.

Becoming an *authentic practitioner* of DPI is only learned through intense training and supervision. Most optimal CBASP practitioners had to un-learn many professionally trained behaviors that taught them to maintain interpersonal distance. These habits were replaced with more reciprocally interpersonal DPI patterns which they mastered.

Several personal requisites which the best CBASP therapists exhibit are discussed below. (1) One must know oneself emotionally. *Emotional maturity*, sometimes achieved by CBASP psychotherapists through a personal therapy or clinical supervision, is a *sine qua non* requirement for DPI administration. This includes being aware of one's interpersonal and cognitive reactions to patients, being able “to track” (self-monitor) one's feelings and thoughts moment-to-moment during the session, and possessing the skills to impart these reactions in ways that facilitate the patient's well-being. Having the skills to identify the interpersonal impacts patients have on practitioners (28–30, 34) enables the individual to utilize these impacts via verbal and non-verbal feedback in a disciplined and salubrious way. (2) The second requisite is *giving oneself permission to be oneself with the patient*. Psychiatric and psychological clinical training rarely teach trainees to utilize their emotions with patients. Often, the only trainee-emotions acceptable to supervisors and attendings are *acceptance* and *empathy*. The novelty of CBASP training is that participants are told frankly that they may be themselves with patients, and they are rigorously taught how to use their emotional and cognitive reactions in contingent ways. The difficult hurdle comes next—*they must then give themselves permission to be themselves with patients*. Master CBASP therapists have actualized the self-permission step with aplomb.

(3) *One must overcome the fear of hurting patients by being oneself*. Since all of us have been trained under the aegis of the personal involvement taboo, most don't know what will happen if they disclose something personal to patients. Many professionals I've trained are frankly afraid that expressing personal reactions in contingent ways will hurt patients and jeopardize their effectiveness. There are also some practitioners who for various reasons don't want to disclose or express their emotions—DPI is clearly not for them. Optimal CBASP practitioners who have taken the risk and are able to utilize their patient reactions in contingent ways have discovered that DPI is a robust vehicle for modifying maladaptive behavior. (4) Lastly, *the core word in DPI is “disciplined.”* The *cardinal rule* of DPI is that one must never do anything to hurt the patient. The well-being of the patient is primary! CBASP therapists pay close attention to any negative side effects that may accrue from their interventions. I have never known of a case where a successful CBASP practitioner willfully damaged the patient. Conversely, utilizing DPI that offers patients a counter-conditioning relationship with a thoughtful and non-maltreating human being is facilitative and salubrious. Most preoperational patients must be taught to relate interpersonally. The learning is best imparted in the trenches of interaction with a personally involved and disciplined CBASP teacher.

### Able to Implement an Acquisition-Learning Approach to Therapeutic Administration

**Figure 1:**
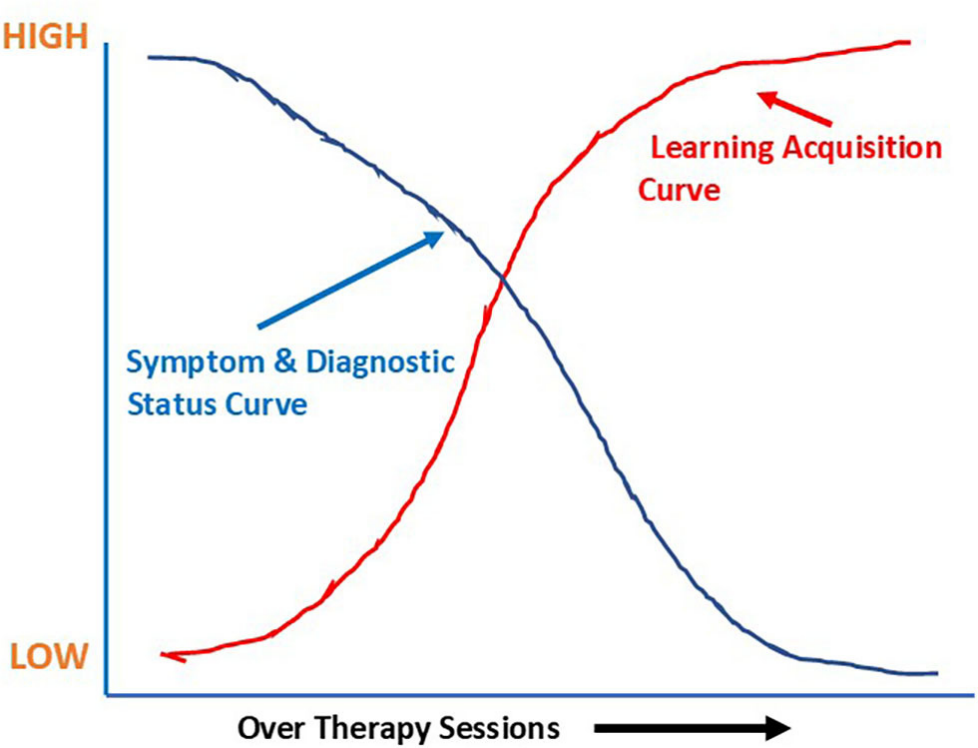
CBASP acquisition learning and symptom assumption curves shown in a hypothetical design space.

CBASP is an operationalized model of psychotherapy and the two major operationalized goals of treatment, *felt dyadic safety* and *perceived functionality*, must be acquired over the process of therapy. CBASP measures in-session learning as a primary means for determining treatment effectiveness. The major acquisition learning assumption is stated in the following manner: *If one learns what the CBASP model teaches, disorder management will be achieved* (6, 10). This assumption is illustrated in the hypothetical design space shown in Figure 1. Figure 1 illustrates what happens to the symptom measures when patients learn the tasks of treatment.

**Figure 2:**
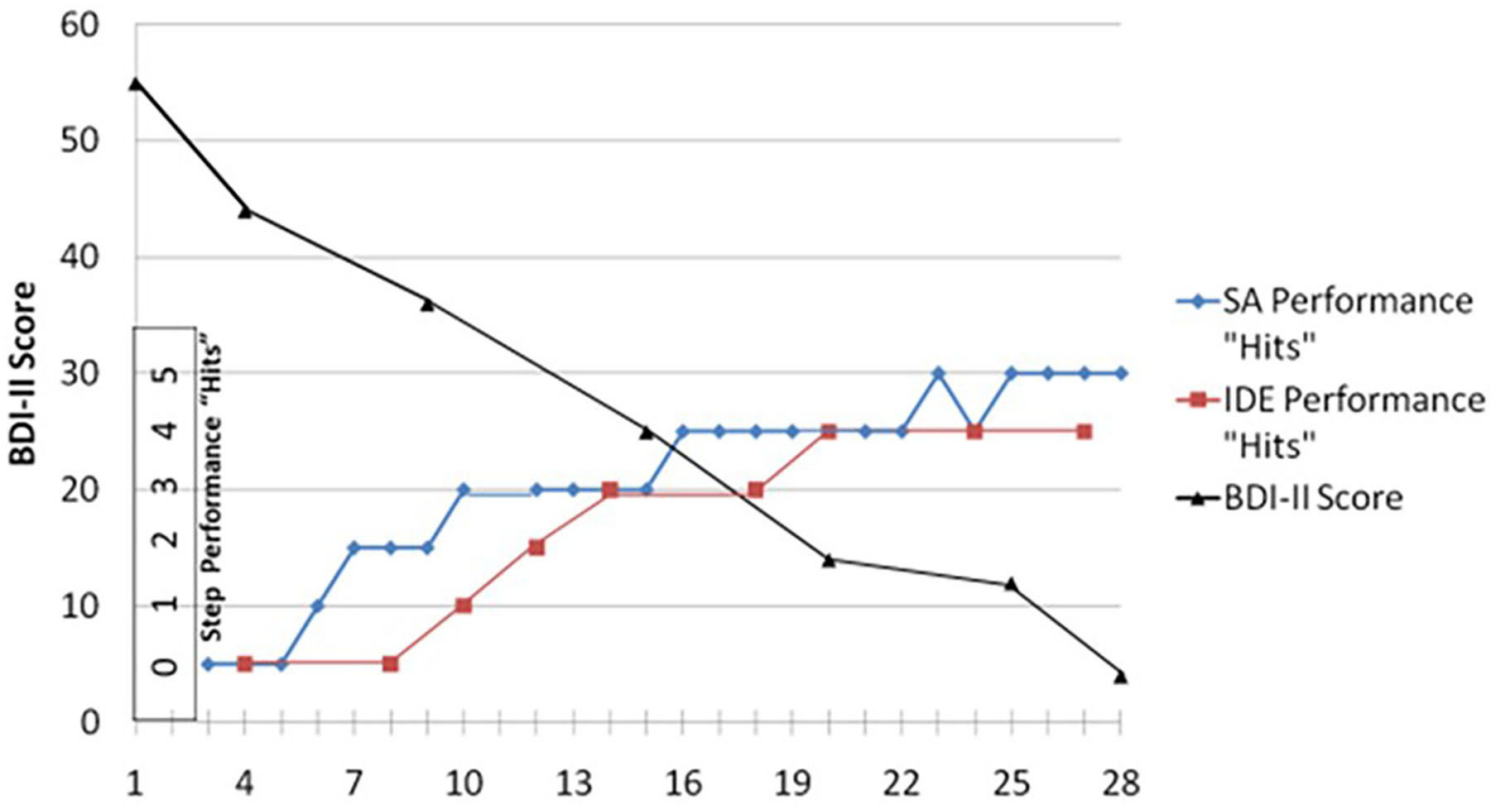
Sandra's BDI-II scores “averaged” every fifth session: her SA step performance “hits” using the Patient Performance Rating Forum; and her IDE step performance “hits” using the IDE-Rating Forum. Both were rated by a clinical rater.

Figure 2 illustrates data taken from the case of Sandra where she performs to criterion the two major learning goals of treatment (i.e., achieving felt *dyadic safety* with the IDE and achieving *perceived functionality* with the SA), and we observe a progressive decrease in one symptom measure [i.e., Beck Depression Inventory-II (35)].

One word of caution must be stated here. It is the author's firm belief that early-onset PDD is *never* fully cured. CBASP therapy is an endeavor striving to educate patients how to manage a lifetime disorder. Various forms of maintenance treatment will also be required after the weekly sessions end [e.g., (18, 36–38)]. Patients should be informed from the outset that their disorder is not curable but highly manageable (6); the lessons learned in psychotherapy, if one is to avoid further periods of depression, must be practiced daily for the remainder of one's life. PDD falls into a category similar to two other lifetime disorders: namely, *diabetes mellitus*, a metabolic disease produced by a systematic failure to adequately regulate blood sugar levels, and *hypertension*, a cardiovascular chronic medical condition in which the systemic arterial blood pressure is elevated. Both these physical disorders can be controlled and maintained by proper prophylactic behaviors—so can chronic depression. Failure to practice good preventive maintenance after treatment ends may lead to death in the case of the two physical disorders and to relapse and recurrence in the case of chronic depression. Achieving criterion performance with the two goals of treatment and generalizing the in-session gains to the daily living arena launch the patient into the post-therapy phase.

*The Two Goals of CBASP Therapy*. Premier CBASP practitioners approach their cases as learning endeavors and define their clinical role as “teachers.” The prediction stated above that mastery of the goals of CBASP resolves the chronic disorder needs to be elaborated. The prediction suggests that the psychopathology of early-onset PDD is maintained because of two problem-variables addressed by the two major counter-conditioning treatment goals of CBASP.

The first treatment goal involves (1) *teaching the patient to experience “felt safety” with the clinician, meaning that patients acquire the ability to successfully discriminate the clinician from maltreating significant others*. Bouton (39) argues that when behavioral avoidance is present, fear is motivating it. The patient's interpersonal avoidance has long been conditioned by a fear of interpersonal encounter. The fear is well-learned and derives from a toxic family arena where significant others have hurt the patient. This fear and the subsequent social avoidance that accrues, taken together, prevented the individual from participating in normal adolescent social encounter which is a requisite for normal teen-age development. The inability to have learned the social lessons of adolescence due to interpersonal avoidance have come at a high price; it has left the patient unable to function adaptively with others. The interpersonal fear-avoidance is addressed in the first goal. The therapist, actualizing the DPI relationship and specifically teaching the person to correctly self-administer the Interpersonal Discrimination Exercise (IDE) (6, 10), teaches the individual to discriminate between maltreating significant others and the practitioner. Criterion performance in the self-administration of the IDE suggests that the creation of a *felt safety zone* has been achieved within the session—the first goal in successful CBASP therapy. More will be said about the IDE in the techniques section to follow.

The second problem-variable is addressed by the second treatment goal. The goal is stated as follows: (2) *Patients must learn to recognize the interpersonal consequences of their behavior as evidenced by the correct self-administration of the Situational Analysis (SA) exercise*. The achievement of the criterion performance in the self-administration of SA is labeled *perceived functionality*. As noted previously, an early maltreatment history exerts pernicious social effects on the patient. Patients, to survive the hell of the family and for self-protection, perceptually disconnect themselves from others to avoid hurtful social encounters. They erect interpersonal walls of isolation behind which they live in solitary confinement. *The most disastrous result of this self-protectionist strategy is that the chronic patient's perceptual disconnection from the social environment effectively removes the person from the social arena and precludes one from being informed by interpersonal feedback*. In short, the person now lives without a social environment. The environment has lost its shaping power to influence and left the individual in a trajectory of “isolated sameness.” One consequence is that the perception of TIME stops for the patient as the *present* denotes only a replay of the *past* and the *future* bodes only more of the same. SA is designed to perceptually connect CBASP patients with their social environment so that interpersonal feedback can begin to shape behavior. This *person x environment* connection must occur first with the clinician. *Perceived functionality* means that the person x environment connection has been achieved. More will be said about SA in the techniques section to follow.

Summarily, good CBASP therapists are able to establish an arena of dyadic felt safety and perceptually connect their patients to themselves and others. With interpersonal fear now pushed aside, avoidance is diminished, and the patient is better equipped to learn the lessons of CBASP.

### Able to Adhere to the Standards of CBASP Technique Administration

In this section, the techniques described will be the Significant Other History (SOH), Transference Hypothesis (TH) construction, the Interpersonal Discrimination Exercise (IDE), and Situational Analysis (SA). The best CBASP practitioners I've known become artists when it comes to administering these techniques. Relying on the clinical role of Disciplined Personal Involvement (DPI) and with a sound knowledge of the methods, they administer the techniques the way they were designed to be administered. These clinicians always remain cognizant of the two goals of the model which are the creation of in-session dyadic safety and secondly, helping patients learn to recognize the consequences they produce on the therapist as well as on others. The best practitioners I've worked with have also learned “to rely” on the model procedures to do the essential work of treatment.

This point cannot be made strongly enough: Administering a case and relying on the techniques of CBASP and its approach to PDD psychopathology is very different compared to working with the chronic patient and relying on other personal strategies of change such as being a caring personality, being empathic and nurturant, and providing unconditional positive regard. Not only is the DPI clinical role lost in the administration of these alternative approaches, acquisition learning and the focus of treatment are also compromised as they subtly shift the focus of the consequation of patient behavior, the main focus of treatment, to other personal role activities.

*a) Significant Other History (SOH)*. Capable CBASP therapists use the SOH to elicit information identifying the patient's early abuse history and the maltreating significant others who administered the abuse. *Significant Others* denote the major players in the patient's life, persons who have influenced the individual to be who they are or informed the direction their life has taken. The SOH is administered in the second session. Developmental events with toxic significant others shape expectancies about what is likely to happen in psychotherapy. Knowledge of these negative injurious patient expectancies enables clinicians to identify potential relational *hot spots*. CBASP practitioners, who use the SOH wisely, become cognizant of the historical factors that contribute to the PDD disorder and, using this information, they avoid interpersonal rupture events that may fatally undermine the dyadic relationship.

An example will illustrate this point. A CBASP trainee, Tom, was overly helpful to everyone and always extended himself in nice gestures to patients and colleagues alike. Tom began his first session with a 21-year-old female patient. He was making coffee and when the patient entered his office, he offered her a cup of his newly brewed café-au-lait. The offer was extended with his usual kind demeanor. The sexual abuse history of his patient was not known to Tom. Her biological father had engaged in sexual relations with her for several years. The father, always when his wife was absent from home, would become very nice and solicitous of her needs, and then would begin his sexual advances which always ended in intercourse. The kind actions of Tom awakened her learned expectancy of what was coming next—she bolted from the room. Luckily, she returned to treatment and Tom then understood what precipitated the departure. Behaving in his usual kind way, without benefit of the SOH, had had disastrous effects. With the SOH, good CBASP therapists do not “fly blind” with patients.

The interpersonal core fears of patients are identified with the SOH. Patients often enter treatment fearful of specific negative reactions from therapists when they behave in certain ways. The SOH pinpoints many fearful expectancies which may occur in four domains. The domains are the following: (1) *relational intimacy*; (2) *behavioral disclosures* of needs or highly personal content; (3) *mistakes* the patient makes during treatment; and (4) *negative emotions* patients feel toward therapists. To identify the most salient core fear, skilled CBASP clinicians identify the dominant domain that emerges as they proceed through the significant other history list. The interpersonal expectancy is that their clinician will react like hurtful significant others did (note the above example with Tom).

*b) Transference Hypothesis Construction (TH)*. Excellent CBASP therapists productively use the recommended number of TH interventions in about 30% of the sessions (40) to help patients make an interpersonal discrimination between maltreating significant others and themselves. After reviewing all the information derived from the SOH in session two, practitioners construct a one-sentence TH. The sentence makes explicit the patient's core fear event and what consequences are likely to follow if this event occurs in the session. For example, if the “relational intimacy” domain is implicated as the salient core fear, the TH might be the following: *If* I (Joe, the patient) become interpersonally close with Bill (my therapist), *then* Bill will begin to point out my mistakes and weaknesses and tell me what a loser I am (the way my significant other *father* did). Notice that the TH sentence first identifies the fear event (relational closeness), and then spells out the expected consequence (derision and rejection). Whenever interactions enter the “relational intimacy” domain specified by the TH, the most effective CBASP clinicians will know that they are in *hot spot* territory. The patient will then be asked to discriminate between the consequences that accrued with *father closeness* and then the consequences of the closeness with the therapist. This task brings us to the next technique, the Interpersonal Discrimination Exercise (IDE), which is related to the first goal of CBASP (i.e., creating a dyadic safety zone).

*c) Interpersonal Discrimination Exercise (IDE)*. The IDE is designed to interrupt the patient's orbit of “sameness” and focus the person's attention on the novel behavior of the practitioner. In the beginning of treatment, the behavior of clinicians is mistakenly perceived as being no different from that of maltreating significant others. This misperception must be revised, and optimal CBASP therapists use the IDE as the corrective tool.

The IDE, administered as the clinician and patient enter a *hot spot* zone, is a four-step exercise that asks four questions in this order:

- What would your significant other (SO) have done when you said or did this? (core fear event)
- What did I just do when you said or did this?
- *Now, compare and contrast my behavior with that of your SO*.
- If I turn out to be different than your SO(s), what are the implications for you in this relationship?

Patients learn to self-administer this four-step discrimination exercise without assistance from the clinician. Mastery of this goal is designed to drive a perceived wedge between the behavior of toxic significant others and the therapist. If the discrimination is not made explicit using the IDE, PDD patients will not make these distinctions. Acquiring these discriminations is not easy and requires repeated IDE trials. It cannot be achieved in one administration of the IDE. These erroneous perceptions are so entrenched in the brain's “granite memory system” that in 2000 (p. xxiv), McCullough wrote a description of what modifying them is like:

*Treating the chronically depressed adult, dislodging the refractory cognitive-emotional and behavioral armor that is the disorder, is analogous to breaking through a granite wall using a ten-pound sledgehammer. One hits the wall repeatedly in the same area with little or no effect until, almost imperceptibly, a slight hairline crack appears. Under continuous pounding, the crack gradually enlarges until, finally, the wall breaks and crumbles*.

As noted earlier, the goal of IDE mastery and the first goal of CBASP is the creation of felt safety on the part of the patient. Able CBASP clinicians utilize the IDE to help patients extinguish these confining perceptions of the way life has had to be and frees them, in a safe interpersonal arena, to learn how to behave within new horizons of interpersonal relationship.

*d) Situational Analysis (SA)*.Situational Analysis, a five-step exercise that patients will learn to self-administer, is designed to achieve the second goal of CBASP. That goal is to perceptually connect patients with their social environments so that the way they behave is informed by the therapist first, and then by others. In contrast to the operant functional analysis of behavior methodology (41, 42), SA teaches patients to cognitively identify/recognize the consequences of their behavior in contrast to the identification of behavioral consequences achieved through experimental reinforcement manipulation. The SA exercise also keeps patients in a *participant role* instead of talking about themselves in an observer role. The SA exercise is also the most difficult CBASP technique to learn. I have not seen many CBASP practitioners perform the exercise to perfection. When able clinicians administer SA correctly, it becomes high drama in the session as patients begin to learn that their behavior has consequences.

At the outset of treatment, most PDD patients do not understand that they produce the misery of which they complain because they are not social-interpersonal abstract thinkers. SA is designed to demonstrate tangibly in the session the consequences of behavior. Therapists do not talk about what patients do nor do they cajole the person into behaving otherwise; clinicians do not use logic to suggest alternative strategies, and they do not verbally punish the individual for behaving foolishly. SA is a learning exercise *that shows, illustrates, and demonstrates* visibly and auditorily the interpersonal consequences of one's behavior. Chronic patients can “talk about” themselves from an observer perspective forever and never change anything. Therapists who ask observer questions keep the patient in a non-participant “neutral holding pattern,” and the best clinicians know that observer questions are a waste of time for this patient. Examples of some observer questions which preclude one from having to participate in behavioral consequences are the following:

- “*How are you feeling right now?”*
- “*Why do you think you did this?”*
- “*Why do others react to you this way?”*
- “*What do you think your stimulus value is right now?”*
- “*What does the other person make you want to do?”*
- “*Why do you want to do these things?”*
- “*What effects do you have on others?”*
- “*What were you feeling when you did this?”*
- “*How might you behave differently?”*
- “*How did Patricia make you feel?”*
- “*Why do you think you stay depressed?”*
- “*Do you ever want to change and do things differently?”*
- “*Where did you learn to behave this way?”*

Optimal CBASP clinicians who administer treatment from a DPI perspective maintain a high-level of personal encounter, do not ask patients to “talk about” themselves, and potentiate participant encounters with SA. They are also mindful that the preoperational patient is easily confused with too much information at one time. That is why the best CBASP therapists keep SA simple and the patient highly focused during the exercise. Good therapists want their patients to learn to self-administer SA, that is why their administrational simplicity is so prominent. Watching these practitioners work is observing an art form taking shape. From personal experience over the years, the more the author has administered SA, the simpler his exercises have become and the more he highlights behavioral consequences.

The SA methodology teaches global-thinking patients to focus on one problem at a time. Many begin treatment complaining that they have so many problems, they don't see how focusing on one will do them any good. Despite this protest, patients learn to describe one situational event *(a slice of time)* occurring between the patient and another person (Situational Description: Step 1). The event must have a discrete beginning point in time, an endpoint that can be behaviorally observed, and some brief story in between. Next, one to three *interpretations* are requested which expose what the event meant to the individual. The interpretations or reads must be stated in one brief sentence (i.e., The event meant “blank”) (Interpretation: Step 2). Third, patients describe how they behaved in the interpersonal situation (i.e., tone of their voice, their non-verbal expressions, the actual words they said, etc.) (Behavioral Description: Step 3). Fourthly, the individual, in one sentence, describes the endpoint or how the slice of time turned out. This step is called the Actual Outcome or the situational consequence (Actual Outcome, AO: Step 4). The final step asks patients to state in one sentence, how they would have liked the situation to have turned out. This step is called the Desired Outcome (DO) and in SA, the DO becomes the *situational goal* and *motivational component* of the exercise (Desired Outcome: Step 5). Many patients, having never thought about what they wanted nor set their desires as a behavioral goal, need considerable assistance in the beginning to construct a DO sentence. Patients are encouraged to frame the DO as something they could have done or said and avoid positing a DO in the social environment (e.g. “*I wanted her to like what I had done”* vs. “*To ask her if she approved of my behavior.”*).

Desired Outcomes are rarely achieved in early SA administrations; rather, mismanaging interpersonal situations and not achieving one's DO are usually the norm, and this pattern becomes evident during the exercise. *Remember the goal of SA: to illustrate to the patient the consequences of their behavior*. Patients, in being bound within the slice of time and not allowed to move into global thinking (e.g., “No one likes me;” “Nothing will ever work out for me;” etc.), have to confront their cognitive and behavioral errors (in the presence of the therapist) that resulted in a poor Actual Outcome—and one that was not equivalent to their DO. Said another way, they didn't get what they wanted when the AO ≠ DO. Rarely has the patient ever confronted the consequences of their behavior, particularly when the consequences were not desirable. It is an anxiety-evoking experience but sets the motivational wheels in motion for change. SA leaves the burden of change in the patient's court. If they want to achieve their DOs, they will have to change their behavior. If nothing changes, then their DOs remain unattained—not a pleasant state-of-affairs. Exceptional clinicians can tolerate high levels of patient anxiety and by not decreasing patient discomfort with reduction strategies (e.g., “You'll do better next time,” etc.), the stage is set for the patient to reduce their own anxiety by enacting more adaptive behavior.

Over time, patients learn to work within the small “slice of time” by using abstractive thought. For example, they begin to think *about* alternative things they could have done. They must think *about* what they want. They must think *about* others in realistic ways. They must *evaluate* their problem-solving efficacy in the slice of time and *self-correct* their mistakes. All these strategies require abstractive thinking—an ability the patient did not possess when therapy began. As they move toward mastery of SA, they look at themselves, others, and their social environment in alternative ways—all this entails abstractive thought. The upshot is that new interpersonal possibilities are now open and can be seriously considered. This new thinking counters the old negative preoperational thinking (i.e., *The way it is, is the way it must be*).

### Able to Implement Several Facilitative Interpersonal Skills

- *Introduction*. Over the years, the best CBASP psychotherapists I've observed move from session-to-session almost seamlessly and always appear to react to patients in appropriate ways. They are also keenly aware of what's happened between themselves and the patient in previous sessions which enhances the continuity of treatment—they “bridge” the past with the present with little effort. These individuals choreograph contingencies to reinforce adaptive behavior when it arises—they just seem to know when the patient has made an adaptive move even when the behavior might appear to others small and insignificant. They catch it, make the behavior explicit and consequate it with reinforcing acknowledgment! In addition, they have a solid grasp of where patients are in terms of the CBASP learning goals and what steps in the IDE and SA need extra attention. Knowing the personal idiosyncrasies of the patient and where the end-goals of the case are, where the process of therapy stands in the present, and how much remains to be done to reach the goals of treatment characterize the work of optimal practitioners. These individuals actualize skills that I, frankly, do not know how to teach. Where does this quality performance come from? I cannot say it comes from clinical experience because I've seen seasoned veterans who do not achieve this quality in their work—they may be quite good and successful with chronically depressed patients, but there is a difference in their work-quality and it is observable.
- *Authentic Disciplined Personal Involvement*. Some CBASP therapists I have known are *authentic* human beings. That is, they are real and genuine persons who don't practice psychotherapy *playing out* an interpersonal role that is not who they are. I've heard patients describe such individuals this way: “*What you see is what you get.”* They don't have to be nice; they don't have to be accepting, caring or nurturing; but they can be nice, accepting, caring and nurturing if it's in the best interest of patients. They are themselves with patients, and, over the course of therapy, patients learn to relate to an honest and genuine human being who doesn't play professionally-learned therapy games. I once knew a practitioner who threw up in his office trash can in full view of the patient. I asked him why he didn't excuse himself and go to the bathroom. He told me that the patient had just disclosed a horrific sexual abuse story that nauseated him. He wanted the patient to see, first-hand, his reaction to what had happened to her.
- *Exceptional CBASP clinicians feel comfortable with the chronic condition*. Not everyone works well with chronic conditions. Some like quick change and feel most comfortable moving on to the next thing. This is not possible for those who treat the early-onset PDD patient. Nothing changes quickly. Therapy moves slowly, new learning is acquired sluggishly, old perceptual and behavioral habits die hard, and clinicians must be willing to remain in the trench for the long haul. The best therapists are patient and understand the slowness that new learning requires and how much time it takes to achieve the extended processes of extinction. “Start and stop, start and stop and then, begin again”—it is an apt description of the challenge clinicians face who treat the chronic patient. Feeling comfortable with everything that working with chronicity entails, being able to tolerate the frustration and disappointment with patient failures all the while continuing to remain hopeful is only for a few courageous souls. I can spot those who feel comfortable with the chronic individual by the way they talk about patients. They evince patience and an explicit understanding of what is required to modify refractory behavior. Quite frankly, they are as tough as their patients are.
- *The best therapists “trust” in the CBASP methodology*. This characteristic does not mean the person is “slavish” when it comes to the rules of technique administration. It means that the CBASP technique protocols will be administered by the “spirit of the Law” and not by the “letter of the Law” and the rules will be tailored to the patient's idiosyncrasies. The CBASP guidelines for technique administration provide a reliable roadmap delineating what needs to be done first, second, and so on, and optimal therapists count on the technique roadmaps for strategic direction. I've listened to many non-CBASP clinicians talk about treating chronic patients. They frequently talk like they have to start over with each new case—they have no proven process precedents to rely on, to fall back on, and to guide them. The exceptional CBASP therapist knows where to start, what must be done, and what the end-point goals are. There is no starting over with a new case. The CBASP roadmap protocol spells out the therapy trajectory and practitioners trust the map for guidance over the twists and turns of the case.
- *The best CBASP therapists are talented acquisition learning teachers of the model*. The lesson plans are the protocols for CBASP administration; that is, teaching SA and the IDE to criterion as well as teaching assertive behavior so that patients may achieve their situational Desired Outcomes. The best teachers can effectively shape behavior and teach by small steps (31). Shaping mean being able to conceptualize behavioral goals in increments of learning—a skill that requires thinking small, breaking down the entire learning program (like SA) in small sequential steps, and being able to pinpoint what must be learned first, second, etc. Only later will the entire learning program be mastered. The learning acquisition approach to doing psychotherapy makes the CBASP model unique in the psychological and psychiatric field. Exceptional CBASP clinicians approach treatment as “teachers” whose primary mission is to teach a salubrious strategy which will enable patients to manage their chronic disorder for the remainder of their lives.
- *Optimal CBASP clinicians verbally “control” the session*. Not being able to gain verbal control of patients precludes one from doing CBASP psychotherapy. I once worked with an analytically trained individual who let patients talk for 45” at a stretch without saying anything. No learning took place, and he and I finally agreed that CBASP was not for him. Good CBASP clinicians gain verbal control and guide the dyadic flow without being overly dominant or rude. They can effectively teach patients to talk in a dialogic manner. One of the interpersonal goals is learning to talk with the therapist reciprocally; this means, talking when appropriate, answering questions when asked, asking questions when the need arises, and remaining silent and listening attentively when spoken to. Individuals cannot learn if verbal control is absent. Many individuals enter treatment having never been listened to or taken seriously—they expect therapists to behave just like maltreating significant others.

Learning is not possible if the clinician has not obtained verbal control of the patient. This is not always easy to achieve, but until verbal control is established, the practitioner does not have a workable case. Some patients cry for most of the hour, others talk non-stop, some never say a word, some change the subject frequently, others refuse to make eye contact and instead look out the window, and a few complain endlessly that they fail at everything. Obtaining verbal control of the patient is the first thing that must be achieved before CBASP treatment commences. The best therapists work effectively with this obstacle and achieve the control they need. Then and only then, can CBASP treatment begin with one who is now in an optimal learning mode.

g) *Exceptional therapists have Interpersonal flexibility treating two modal types of chronically depressed patients—that is, (1) physically and sexually abused persons and (2) emotional and physically deprived patients*. These two patient types require different DPI styles. The physically, emotionally, and sexually abused individual needs a practitioner who can “hold back” in their reactions as they have already been over-powered by significant others who have hurt them. A gentle approach is called for which means the practitioner must tread lightly rather than rush in with queries or emotional reactions—such patients have already been the recipients of persons running over them in interpersonal encounter. Conversely, the physically/emotionally deprived patient will require therapist behavior that “moves in” and does not hold back. Such persons usually come to treatment expecting nothing to happen or little or no response from the practitioner. It is up to the therapist to see that these expectations are not fulfilled. Their developmental environments were devoid of caring and attention-giving and they were mostly left alone to fend for themselves. No one knew of their scholastic accomplishments, or athletic heroics, or what they needed emotionally or physically. They grew up in a world by themselves expecting nothing from others. The most gifted CBASP therapists have the interpersonal flexibility to respond differentially to these two individuals providing support to the notion that patient diversity means that “one size does not fit all.”
h) *Talented CBASP clinicians can tolerate “silent periods.”* Silence in the session may be anxiety-provoking for some therapists. As happens in those instances, therapists reduce their own felt discomfort by initiating more verbal discourse. Mature practitioners tolerate the discomfort of silence when it arises and use it to the patient's advantage. Silence is a “time for reflection—*where have we been and where are we now?*” It may be a time to identify what's prompted the stopping point but not to terminate it too quickly to make oneself feel better. If something blatantly obvious has happened between the interactants, time may be needed for the patient to recover. If clinicians are unsure about what interrupted the conversational flow, after an appropriate time has passed, they can ask patients to clarify the silence. Or they can just wait and see what happens. For the most mature clinicians, silences frequently yield productive dividends.
i) *Optimal therapists can manage anger in the session*. Anger is one of the most difficult emotions for mental health practitioners to deal with. Therapists usually react in one of three ways: (1) they work harder, (2) they interpersonally withdraw, or (3) they counter-aggress. None of these strategies are effective. The most effective tactic is to identify *why* the patient has pushed the therapist away—what is precipitating the hostile reaction? Kiesler (28, 29) opines that anger or hostility is an interpersonal impact that communicates: “Get away from me;” “Get out of my face!” One clinical psychology trainee was working with a very hostile patient who continued to denigrate his performance making the trainee feel incompetent. The trainee wanted to transfer the case because his Rogerian “unconditional acceptance” tactics were not working. I asked if he wanted to learn how to deal with anger. He said, “Yes!” The strategy he subsequently employed was directed toward identifying the source of the patient's anger. He began to ask his patient questions like the following: “Why are you beating up on me?” “Why do you keep punching me in the face?” The literal nature of these queries more often than not evoke surprise reactions as well as some verbal responses such as “I'm not doing that!” or other types of protest (e.g., “You ought to be able to handle my anger;” “You should have been trained to deal with such reactions.”). Then, a more honest reply often follows. He confided his fears of relating to men and to maintain a safe distance, he always fought. The trainee's therapy then began to move in more profitable directions. If the therapist had not personally raised these questions, the causes of the anger might not have been addressed and more adaptive interpersonal strategies might never be learned. Optimal practitioners manage the hostile emotions of their patients by teaching them other ways to interpersonally relate.
j) *The best CBASP clinicians can tolerate their anxiety without reducing it*. All of us become anxious or uneasy when certain behaviors are emitted or when patients bring up particular topics. Exceptional therapists stand fast and do not change the subject to reduce their discomfort. The reward is that they can help individuals address the problematical areas that have been put on the table. It is not an easy challenge to master, for anxiety is painful, uncomfortable, and potentially fear-provoking. Examples might be not knowing what to do or say, being confused by the patient's behavior or comments and not knowing how to respond, reacting with anxiety when one mentions certain subjects or topics such as relational intimacy, hearing patients disclose that the therapist has disappointed or angered one by some comment or reaction, faced with a request for a hug or embrace, or listening to a story that awakens old anxieties about past experiences. What to do? The best CBASP therapists tell us to stop and ask oneself what the patient needs right now. Stop and identify where the source of the alarm is and then consider the practitioner's Desired Outcome in the moment which, hopefully, is in the best interest of the patient. What does the patient need right now and what must I do to deliver what's needed? The word that comes from the experts is the following rule: Stop, Look, and Listen, to myself first and then, to the patient.
k) *The best therapists know how to “walk with” the patient at their pace*. This skill is called “pacing” and the most effective among us walk with the patients we see. How does one learn to pace? There is a rule of thumb that helps. It is as follows: *the patient is always right and right where they ought to be*. It's not the therapist who is right, it's the patient. It's not where the therapist is, it's where the patient is and he or she cannot be anywhere else right then. The ablest clinicians remind us that our job is to identify *where* the patient is moment-to-moment and to recognize *what* is going on. If we can answer both these questions, then we can walk with the individual. If we cannot, we are either walking by ourselves or walking ahead or behind the person. Pacing means finding the learning rate of the person, stopping when necessary, backing up if the situation calls for it, and then, moving once more when the pace is picked up. We walk with the patient—we do not ask the patient to walk with us. How does one teach this skill? If progress halts and change is not forthcoming, we must be thrown on the alert. Are we asking too much too quick or have we neglected to motivate the person? Learning to listen *to the progress* of the individual will help us walk with and avoid pulling and pushing.
l) *The best therapists avoid preaching, exhorting or telling*. The modal statement of the best CBASP therapists is an interrogative one. Asking questions always allows the patient to play their cards first, and then the practitioner knows what and how to respond. Preaching, exhorting, and telling the interpersonal avoidant patient is a waste of breath and an ineffective therapy strategy. Since fear drives avoidance, telling someone what to do or exhorting one to act never extinguishes the fear. The excellent clinicians pinpoint/target the fear and extinguish it first. Then, the avoidance is modified. The fear may stem from skill deficits or from earlier learning where, in certain types of encounter, the patient has always run away. Teaching the individual to take an alternative action instead of running away is what is needed.
m) *The exemplary skill of introspectively tracking the “interpersonal impacts” patients have on practitioners, moment-to-moment, and when appropriate, acting on them, is rare*. This skill involves three things: (1) one must have a sound knowledge of Kieslerian (28) interpersonal theory and more specifically, possess a good working knowledge of the complementarity pulls on Kiesler's *Impact Message Inventory*; (2) clinicians must know that the beginning of sound CBASP practice requires that one be able to track the continuous movement of their emotions and be able to utilize this information to identify what is presently transpiring between the patient and practitioner; and finally, therapists must (3) trust their emotional impact interpretations that move from the verbal/non-verbal behavior of the patient to the clinician, and then make mature decisions about what they will respond to and what they will ignore. I have not known many clinicians who were able to master this skill. Emotional maturity and a sensitive awareness of one's emotional life is essential. I have seen a few practitioners who were able to perform this challenge to perfection. It adds a marvelous continuity and smoothness to the process of treatment.

## Conclusion

We must listen to the best CBASP therapists and learn from them. They can teach all of us how to administer CBASP therapy more effectively. In this paper, I have attempted to delineate the optimal CBASP therapist characteristics to showcase how the most accomplished among us utilize the model to achieve notable outcomes. The unique difficulties of the PDD patient and the difficulties of administering this unique model of psychotherapy make successful outcomes wonderful achievements for those fortunate patients who work with practitioners who have taken the time to be the best they can be.

## Data Availability Statement

The contributions presented in the study are original. Further inquiries can be directed at the corresponding author.

## Author's Note

This article delineates the essential and optimal features of the therapists' role in the Cognitive Behavioral Analysis System of Psychotherapy (CBASP).

## Author Contributions

The author confirms being the sole contributor of this work and has approved it for publication.

## Conflict of Interest

The author declares that the research was conducted in the absence of any commercial or financial relationships that could be construed as a potential conflict of interest.

## Footnotes

^1^Many patients are able to function abstractly in their professional capacity—sometimes brilliantly; however, this skill is not operative in their social-emotional interactions.
